# Isolation and Characterisation of Mesenchymal Stem Cells from Different Regions of the Human Umbilical Cord

**DOI:** 10.1155/2013/916136

**Published:** 2013-07-25

**Authors:** Claire Mennan, Karina Wright, Atanu Bhattacharjee, Birender Balain, James Richardson, Sally Roberts

**Affiliations:** ^1^The Robert Jones and Agnes Hunt Orthopaedic Hospital NHS Foundation Trust & ISTM, Keele University, Oswestry, Shropshire SY10 7AG, UK; ^2^Institute for Orthopaedics, The Robert Jones & Agnes Hunt Orthopaedic Hospital NHS Foundation Trust, Oswestry SY10 7AG, UK

## Abstract

Umbilical cords as a source of stem cells are of increasing interest for cell therapies as they present little ethical consideration and are reported to contain immune privileged cells which may be suitable for allogeneic based therapies. Mesenchymal stem cells (MSCs) sourced from several different cord regions, including artery, vein, cord lining, and Wharton's jelly, are described in the literature. However, no one study has yet isolated and characterised MSCs from all regions of the same cord to determine the most suitable cells for cell based therapeutics.

## 1. Introduction

The human umbilical cord (UC) contains distinct anatomical regions comprising two umbilical arteries, umbilical vein, cord lining, and Wharton's jelly (the tissue which surrounds and supports the blood vessels). Each of these regions has been described previously in the literature as giving rise to a great number of fibroblastoid MSCs [1]. Cells isolated from arteries, vein, cord lining and Wharton's jelly have all been shown to be plastic adherent and to be multipotent, differentiating into many cell types such as osteoblasts, adipocytes, chondrocytes, hepatocytes, and neural and cardiac cells [2–4]. They also express markers typical of bone marrow derived MSC (bMSC) [5–7], whilst being negative for haematopoietic and macrophage markers. 

For many years, bone marrow has been considered the “gold standard” for the derivation of MSCs for human stem cell engineering. However, extraction comes with ethical constraint and problems associated with painful harvesting and donor site morbidity. UCs appear to show potential as a source of MSCs for a number of reasons; they are considered medical waste, and therefore their use in research has little ethical concern; they proliferate rapidly in culture and are thought to be immune privileged [2, 6]. In addition, bMSC have shown notable changes with increasing patient age, such as reduction in available cell number, longer doubling times, and lower differentiation potential *in vitro* [8, 9]. Like bMSC, UC MSCs are thought to have an immune privileged status and an immunomodulatory phenotype capable of suppressing the immune response *in vitro* [6], which makes them an attractive candidate for allogeneic cell based therapies.

UC MSCs offer a favourable source of primitive MSCs that can be cryogenically stored in cell banks, thawed and expanded for therapeutic uses. Although many studies characterising cell populations arising from specific cord regions have been reported [7, 10–14], there are no known single studies which have aimed to isolate, culture, and characterise cells from all cord regions from the same cord. 

Many review articles draw together comparisons of characterised cell populations isolated from different cord regions (15, 16, and 17) and report many differences as well as similarities between these cell populations. The authors of these articles often draw attention to the fact that many of the differences could be attributable to variation in isolation or culture technique as well as different handling between research groups. A recent review article states that MSCs isolated from the various compartments of the umbilical cord have yet to be compared with each other [15]. The main focus of the study was therefore to investigate the cell populations arising from four of the distinct anatomical regions of the same human umbilical cord and compare them to a preparation of cells isolated from the whole cord. Therefore providing information on the isolation and culture of a population of cells is worthy of further study and characterisation. We describe for the first time basic characterisation of MSCs obtained from each of the four distinct regions carefully dissected from the same umbilical cord, providing a direct and relevant comparison of cells with the potential for tissue engineering and repair. In addition, we have examined the cell population from an enzymatic digest of a section of complete umbilical cord and compared all populations to bone marrow derived MSCs.

## 2. Materials and Methods

### 2.1. UC Cellular Isolation and Subculture

**Figure 1:**
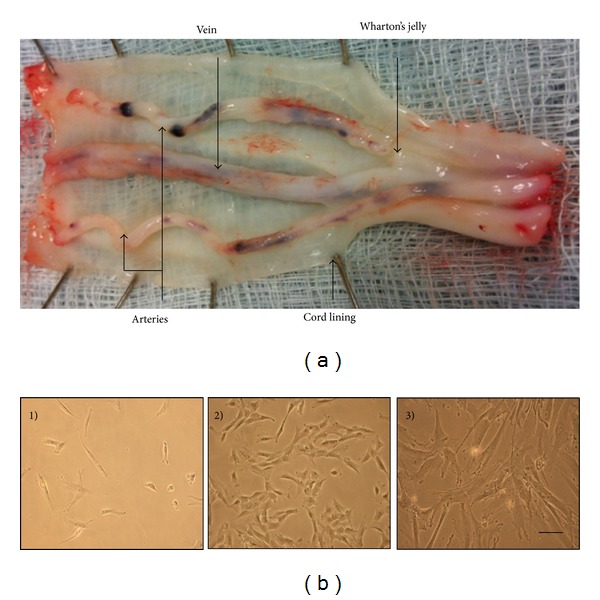
(a) Partially dissected human UC showing umbilical arteries, vein, Wharton's jelly, and cord lining. (b) Umbilical cord cells from mixed cord (and for individual regions) were heterogeneous in morphology when viewed in phase contrast microscopy soon after isolation. This became more uniform with time in culture. (1) P0 ~6 days. (2) P0 ~20 days. (3) P2. Scale bars represent 100 *μ*M.

All samples were obtained after patients' provided informed consent (favourable ethical approval was given by the National Research Ethics Service; 10/H10130/62). UCs were collected from the Robert Jones and Agnes Hunt Orthopaedic Hospital (RJAH) maternity unit and processed within 24 h of natural delivery. Whole cord was washed in sterile phosphate buffered saline (PBS) (Life Sciences, UK) ~three times to remove red blood cells, immersed in 70% ethanol (Sigma, UK) for 30 s, and then immediately washed in PBS before further processing. Approximately 2-3 cm of whole cord was taken for processing as mixed cord, and approximately 6 cm of whole cord was dissected to obtain artery, vein, Wharton's jelly and cord lining (Figure 1(a)). Explant cultures were obtained from each region which was weighed, minced into small pieces (~2 mm^3^) with a sterile scalpel, placed into 6 well plates (Sarstedt, UK), and grown in media containing: Dulbecco's Modified Eagle's Medium (DMEM F12), foetal calf serum (FCS) (10%) (Life Sciences, UK), and penicillin and streptomycin (P/S) (Life Sciences, UK). Tissue explants were removed after 21 days in culture. Adherent cells were passaged upon reaching 70% confluence and reseeded at 5 × 10^3^/cm^2^ in either 25 cm^2^ or 75 cm^2^ tissue culture flasks (Corning Falcon, UK) for growth kinetics or for further culture expansion (resp.). Viable cells were counted by trypan blue (Sigma) exclusion in a haemocytometer. In addition, for mixed cord cultures the whole cord was cut into small pieces (~2 mm^3^) and digested with collagenase (1 mg/mL of type I) (Sigma, UK) for 1 h at 37°C. Tissue was removed from the digest and the supernatant centrifuged at 80 g for 10 min; the pellet was then resuspended in 5 mL of the previous medium and plated in a 25 cm^2^ tissue culture flask. Medium was changed every 2-3 days and cells were maintained in a humidified atmosphere at 5% CO_2_ at 37°C.

In addition human bMSCs were obtained for comparison from bone marrow aspirates or bone chips harvested from the iliac crest of individuals undergoing spinal fusion in the treatment for back pain. Samples used in differentiation studies were from patients aged 29, 40, and 80 years and those used for growth kinetics were from patients aged 34, 42, and 44 years.

Bone chips were perfused with DMEM F12, supplemented with FCS (10%) and (P/S) (1%). Mononuclear cells were isolated by density gradient centrifugation at 900 g for 20 minutes over Lymphoprep (Fresenius Kabi Norge, AS). The buffy coat layer was resuspended in complete media and centrifuged at 750 g for 10 min to pellet white blood cells. The resulting pellet was plated out in DMEM F12, FCS (20%), and P/S (1%) medium (Invitrogen) at a seeding density of 20 × 10^6^ cells per flask. Bone marrow aspirate samples were diluted 1 : 1 with sterile saline and layered over Lymphoprep, centrifuged at 900 g for 20 minutes, and plated as described for UCMSCs. After 24 h, nonadherent cells were removed and adherent cells were cultured in monolayer and maintained in a humidified atmosphere of 5% CO_2_ at 37°C through to passage (P) 2-3 in DMEM F12, FCS (10%), and P/S medium. 

### 2.2. Growth Kinetics Analysis

To calculate doubling time (DT), cells were harvested, counted, and replated when they reached 70% confluency. Doubling time was calculated using the formula DT = (*t*2 − *t*1)ln⁡⁡(2)/ln⁡⁡(*n*2 − *n*1) where *n*2 is the cell number at harvesting, *n*1 is the cell number at plating and, *t*2 and *t*1 are the number of days in culture [16]. A total of four cords were used for growth kinetics analysis and a total of three patients for bMSC. 

### 2.3. Statistics

All data from four cords was normally distributed and differences were calculated using a one-way ANOVA with Bonferroni post hoc test. Levels of significance are indicated *P < 0.05, **P < 0.01.

### 2.4. Immunoprofiling

Flow cytometry was used to assess the MSC immunoprofile of UC cells, using the standard for MSC described by the position paper of the International Society for Cellular Therapy (ISCT) [17]. Cells (P2-3) were harvested, filtered through a cell strainer (70 *μ*M), pelleted, resuspended in 2% bovine serum albumin (BSA in PBS), and counted. One million cells of each population were used for flow cytometry. Cells were stained with directly (phycoerythrin) conjugated antibodies against CD14, CD19, CD31, CD34, CD45, CD90, HLA-DR, CD105 (ImmunoTools, Germany), and CD73 (Becton Dickinson and Company, UK). An appropriate isotype-matched control antibody was used in all analyses. Cells were analysed on FACS scan flow cytometer using Cell Quest Software (Becton Dickinson, UK). Cells from all four cord regions plus the whole cord digest were analysed using flow cytometry for a total of 7 cords. bMSCs from 3 patients were also analysed.

### 2.5. Assessing Differentiation Potential (or Multipotency)

UC cells at P2-3 were assessed for osteogenic, adipogenic, and chondrogenic differentiation potentials. Cells were seeded at a density of 5 × 10^3^/cm^2^ in 6 well plates and grown in monolayer in DMEM F12 and FCS (10%) until reaching ~90% confluency, and at this point the cells were given the appropriate differentiation medium, either osteogenic or adipogenic medium for 21 days. Medium was changed every 2-3 days. Osteogenic differentiation medium contained DMEM F12, FCS (10%), *β*-glycerophosphate (10 mM), dexamethasone (10 nM), and L-ascorbic-acid (50 *μ*M) [18]. Controls were grown in DMEM F12, FCS (10%). Adipogenic differentiation medium contained DMEM F12, FCS (10%), insulin-transferrin-selenium-X (ITS) (1%) (Gibco UK), isobutylmethylxanthine (0.5 *μ*M) (Sigma, UK), dexamethasone (1 *μ*M), and indomethacin (100 *μ*M). Osteogenic and adipogenic controls used media containing DMEM F12, FCS (10%), and P/S. To evaluate the differentiation, in brief, cells were fixed with buffered formalin (10%) in PBS for 10 mins at room temperature and stained with oil red-O for 1 h to assess lipid formation for adipogenesis or naphthol-AS-BI phosphate and fast red for 1 h to assess alkaline phosphatase activity for osteogenesis. A total of 6 cords and 3 bMSC samples (from different patients) were used for differentiation studies. Cells were grown in six well plates (Sarstedt, UK) with three replicate wells from each individual cell population for osteogenic or adipogenic media and three replicate wells as controls per cell population/sample.

A pellet culture system was used to assess chondrogenic differentiation potential. Cells (5 × 10^5^) were centrifuged in a 1.5 mL eppendorf (500 g for 5 min) in 1 mL of chondrogenic medium consisting of DMEM + GlutaMax^TM−1^, FCS (2%), gentamicin (10 *μ*g/mL^−1^), ITS (1%), ascorbic-acid (0.1 mM), dexamethasone (10 nM), and transforming growth factor *β*1 (TGF-*β*1) (10 ng/mL^−1^) (Peprotech, UK). Cells were cultured for 21 days and media changed every 2-3 days. After 21 days, cell pellets were snap frozen in liquid nitrogen and stored at −80°C prior to use. Pellets were sectioned (7 *μ*M) on a cryostat (Bright Instrument Co., Ltd., Huntingdon, UK) onto poly-l-lysine coated slides and stained for glycosaminoglycans (GAGs) with toluidine blue metachromatic stain.

The degree of differentiation was estimated and assessed semiquantitatively by attributing scores ranging from 0 (no staining) to +++++ (very strong staining) of the final preparations.

The scoring system was then given a numerical value ranging from 0 to 5 for statistical analysis using Kruskall-Wallace with Bonferroni post hoc test.

## 3. Results

### 3.1. Dissection of Cord Regions

#### 3.1.1. Expansion of UC MSCs *In Vitro*

**Figure 2:**
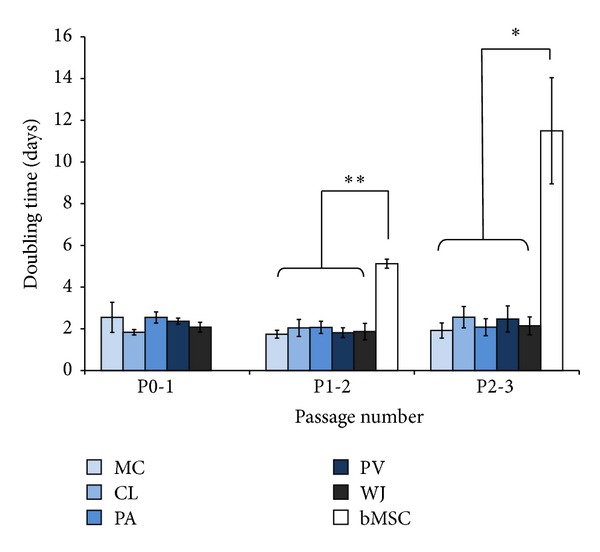
Doubling time (days) of mesenchymal stem cells (MSCs) isolated from different regions of umbilical cord and cells isolated from iliac crest bone marrow at passages 0 to 3. Data shown are means ± SEM for four cords, bMSCs were taken from three patients. Levels of significance are indicated *P < 0.05, **P < 0.01.

Cells from all regions of the umbilical cord and the complete cord digest contained a high number of adherent MSC-like cells which proliferated rapidly in number. Cells isolated from all the cord regions proliferated markedly faster than bMSCs with mean doubling times of 2-3 days at P0 through P3 (Figure 2). bMSCs, in comparison, had a mean doubling time of ~5 days (P1-2) and ~11-12 days (P2-3). UC cells showed a high degree of morphological heterogeneity like bMSCs but most cells had a smaller fibroblastic surface area during early passage (P0) (Figure 1(a)) which developed into a flattened, increased surface area coverage by P2-3.

### 3.2. Flow Cytometry

**Figure 3:**
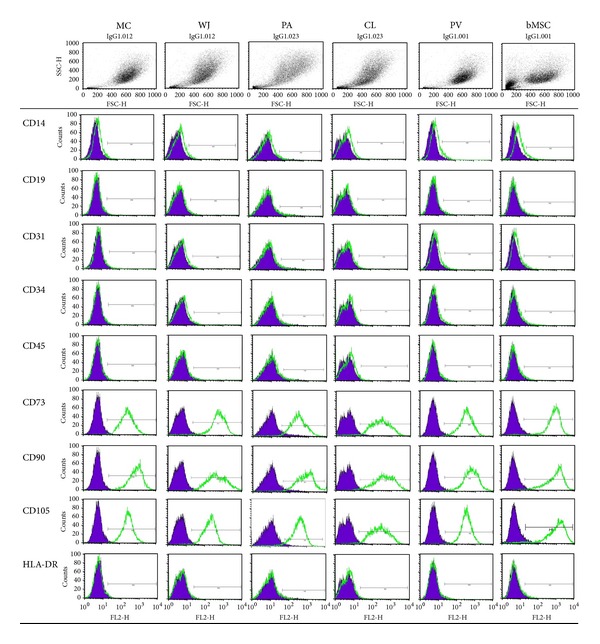
Representative flow cytometry profile of five comparative cell populations isolated from one umbilical cord and bone marrow as required for the International Society for Cellular Therapy (ISCT) definition of MSC. A total of seven cords were analysed in this study along with bMSC from three patients. The purple peak represents the isotype control.

Flow cytometry immunoprofiling demonstrated that all preparations lacked expression of the haematopoetic, macrophage, and endothelial markers: CD14, CD19, CD31, CD34, CD45, and HLA-DR, but remained immunopositive for markers characteristic of MSCs (CD73, CD90, and CD105) (Figure 3).

### 3.3. Differentiation Potential

**Figure 4:**
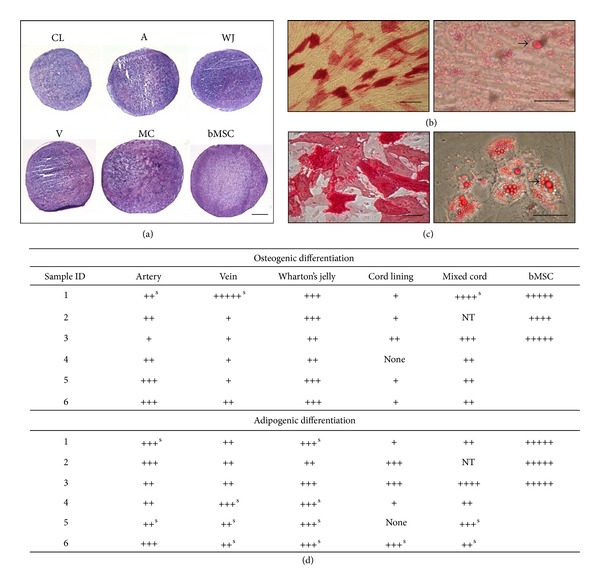
Representative images of (a) chondrogenically-induced cell pellets isolated from different cord regions: cord lining (CL), artery (A), Wharton's jelly (WJ), vein (V), mixed cord (MC), and bMSC. Scale bar represents 1000 *μ*m. (b) Osteogenic and adipogenic differentiated cells from MC. (c) Osteogenic and adipogenic differentiated cells from bMSCs. Scale bars represent 100 *μ*m and 25 *μ*m for osteogenic and adipogenic images respectively. (d) Scoring of osteogenic and adipogenic differentiation of cells isolated from different regions of the umbilical cord and cells isolated from ileac crest. +++++ denotes scoring system given to the best differentiation seen from bMSCs.  ^S^denotes spontaneous differentiation. NT not tested.

The definitive demonstration of multipotency for a cell is the ability to differentiate towards more than one cell type. Cells isolated from all cord regions showed the potential to differentiate to varying degrees as shown by positive osteogenic, adipogenic, and chondrogenic staining (Figure 4). Adipogenesis was seen with positive oil red-O staining and osteogenic differentiation was demonstrated with alkaline phosphatase staining (Figures 4(b) and 4(c)). In addition, toluidine blue stained chondrogenic pellets indicated the presence of GAGs within the pellet matrix although this was seen at different intensities for each cell preparation. 

However, the degree of differentiation of UC cells into the three lineages tested (osteogenesis, adipogenesis, and chondrogenesis) varied between patients and between cord regions. Mixed cord, Wharton's jelly, and artery showed the most consistent differentiation for all of the mesenchymal lineages tested, whilst cells isolated from vein were the most variable (Figure 4(d)). Cord lining showed poor differentiation potential particularly for osteogenesis compared to cells from other cord regions. 

Mixed cord, Wharton's jelly, and artery differentiated consistently down adipogenic and osteogenic lineages. Cells from vein and cord lining showed consistently poor osteogenesis but better adipogenesis in comparison. Lipid droplets which formed in cultures from all cord regions were notably smaller and more cell associated than lipids produced from bMSCs which were both intracellular and extracellular, with lipids observed floating in the media prior to staining. Untreated control cultures, which were grown in regular medium without adipogenic, or osteogenic differentiation stimuli, sometimes exhibited varying degrees of spontaneous fat formation and alkaline phosphatase activity after 21 days of cultivation (Figure 4(d)). Cultures of vein or Wharton's jelly cells showed the greatest propensity for spontaneous differentiation. However, spontaneous differentiation seen in controls tended to be weaker than differentiation staining seen for the cells grown in the differentiation media. The scoring system used in Figure 4(d) was subject to nonparametric statistical analysis since both osteogenic and adipogenic scorings were not normally distributed; Kruskall-Wallace with Bonferroni post hoc test was used to analyse the data. No significant differences were seen between BMSC, MC, and WJ for osteogenesis, but there were significant differences between the following: V and BMSC *P* = 0.007, WJ and CL *P* = 0.01, and CL and BMSC *P* = 0.0004. For adipogenesis significant differences were seen between V and BMSC *P* = 0.01 and CL and BMSC *P* = 0.05.

Cells isolated from all cord regions showed some chondrogenic potential, but there were considerable variations in the intensity of toluidine blue (metachromatic) staining for GAGs between patients and cord regions. Pellet cultures did not demonstrate any one cord region as being consistently better than others for chondrogenic differentiation.

## 4. Discussion

The available data suggests that the UC MSCs present a cell family whose components show various degrees of heterogeneity and multipotency. This study, with standardised procedures for isolation and culture of UC cells, aimed to determine the most efficient means to extract and grow MSCs and to identify the cell population possessing the greatest potential for further study and later developing as an allogeneic source of cells or regenerative medicine.

The isolation and culture of MSCs from individual cord regions including cord vein and the perivascular region [7, 10, 13, 14], Wharton's jelly [11], and cord lining [12] has been reported by many groups. However, none of these studies or indeed any other studies have compared all cord regions from within the same individual [1]. We describe for the first time characterisation of MSCs obtained from each of the four distinct regions carefully dissected from the same umbilical cord, providing a direct and relevant comparison of cells with the potential for tissue engineering and repair. In addition, we have examined the cell population from an enzymatic digest of a section of complete umbilical cord and compared all populations to bone marrow derived MSCs.

Commonly used criteria for defining MSCs are those described by ISCT [17]. In the present study, cells isolated from all cord regions, as well as the whole cord, comply with them, being adherent to plastic and negative for the expression of haematopoietic, macrophage, and endothelial markers (CD14, 19, 31, 34, and 45) but positive for MSC markers, CD73, 90, and 105. Cells were also HLA-DR (MHC class II) negative. In addition, cells isolated from all cord regions differentiated along osteogenic, adipogenic, and chondrogenic lineages but to varying degrees. Results showed that cells isolated from Wharton's jelly and mixed cord had the best osteogenic and adipogenic differentiation potentials overall, but cells isolated from all cord regions showed similar chondrogenic differentiation potential as shown by toluidine blue metachromatic staining of sulphated GAGs (Figure 4(a)). 

Differentiation to osteoblasts and adipocytes was similar to bMSCs isolated from three different patients (Figures 4(b) and 4(c)). Adipogenic differentiation showed that UC-derived MSCs produced small lipid vacuoles in contrast to those of BM-derived MSCs, indicating more mature adipocytes in BM-derived MSCs than in cultures of UCMSC. Other studies report smaller lipid droplets from UCMSC derived from UC blood [19]. BMSCs presented more committed adipocytes (unilocular lipid vacuoles) than induced UCMSCs, perhaps, due to UC MSCs maintaining their multipotency for longer periods *in vitro* than bMSC [20].

Review of the current literature suggests that cells isolated from Wharton's jelly have the best multipotent differentiation potential [1], differentiating into bone, fat, and cartilage, cardiomyocytes [13], neurones [21], muscle cells [22], and hepatocytes [3]. However, perhaps the reason for Wharton's jelly's apparently greatest differentiation potential in comparison to other regions is due to the fact that cells isolated from Wharton's jelly have received the most extensive investigation [1]. We are not aware of a direct and relevant comparison between Wharton's jelly and all other cord derived cells having previously been carried out in the same study until now. 

Although UC cells are referred to as MSCs throughout this study, it is possible that some or indeed all of the isolated cells are in fact pericytes. Experiments have shown that pericytes are located around endothelial cells in the capillaries and microvessels [23], such that they possess antigenic markers typical of MSCs and behave in culture like MSCs [24]. This would be especially relevant to the cell populations isolated from cord artery and cord vein. However, this may also be true of cells isolated from cord lining and Wharton's jelly as it has been demonstrated that MSC-like cells originate from pericytes [19] or that pericytes are indeed MSCs. Like typical MSCs, pericytes grow proficiently in culture, exhibiting the morphology and surface antigens of MSCs (CD73, CD90, and CD105). In addition, pericytes resembled MSCs in terms of developmental potential because the latter differentiate clonally into bone, cartilage, and fat cells when cultured under relevant inductive conditions, which is true of cells isolated from all cord regions in this study. 

This study presents comparative data from four different individual regions of the human UC, as well as the whole UC, in comparison to the more conventional source of MSC from bone marrow. We conclude that MSCs can be found in all cord regions, and that they are morphologically and immunophenotypically similar with the panel of markers used as recommended by the ISCT. The results in this study suggest that the time-consuming and labour-intensive dissection of the cord into discrete regions is not necessary to obtain a valuable population of cells for further study, since mincing sections of whole cord and subsequent enzymatic digestion provides cells with MSC-like properties which are as good as the best individual cord region seen in this study. Extensive culture expansion of these cells could therefore provide a bank of cells which may be suitable for allogeneic cell therapy. This would negate the need for sourcing autologous cells, for example, from adipose tissue or bone marrow, which have associated problems of donor site morbidity and limited cell number.

## 5. Conclusion 

In this study MSCs from four distinct regions of the same cord (artery, vein, Wharton's jelly, and cord lining), in addition to a mixed population of cells from the whole cord, have been isolated and compared for potential musculoskeletal cell therapy. MSCs were cultured from all individual cord regions, as well as enzymatically digested whole cord, demonstrated by their plastic adherence, flow cytometry profile, and ability to differentiate along osteogenic, adipogenic, and chondrogenic lineages. Growth kinetics and MSC immunoprofile showed no significant difference between cells from any of the populations (or isolates). Osteogenic and adipogenic differentiation studies showed variation between cord regions, with the best differentiation seen with Wharton's jelly and whole cord. Chondrogenic differentiation showed little difference between cells isolated from different cord regions.

Enzymatic digestion of the whole cord is easy to undertake, quickly providing a large number of MSC-like cells compared to cells from explant culture of individual cord regions. Cells from whole cord differentiated as well as or better than those isolated from individual cord regions and therefore have potential as a useful source for obtaining promising cell populations for further study.
